# Supplementary material for: Hippocampal damage disrupts the latent decision-making processes underlying approach-avoidance conflict processing in humans
Source: PLoS Biol. 2025 Feb 11;23(2):e3003033. doi: 10.1371/journal.pbio.3003033 (PMC11849986; doi:10.1371/journal.pbio.3003033)
Supplement: Attachment — Submitted filename: PLoS_Biol_Le Duc_et_al_24_rereview.pdf [file pbio.3003033.s002.pdf]

This is a rereview of the manuscript by W. Le Duc and colleagues, which describes a study that investigates the contribution of the hippocampus to approach-avoid decision making in humans.

The revisions have adequately addressed my concerns. Further, the changes made in response to the comments from Referees 1 and 2 combined make the results of the study easier to follow and therefore more impactful. This is a superb study that addresses hippocampal function in a rare human group with relatively selective hippocampal damage; the study reports novel and convincing evidence that hippocampal damage is associated with disruptions to latent decision-making processes during an approach-avoidance conflict task.

I found only one item that needs revision:

1) p. 42 of pdf (p. 27 of ms), 1st para under Between-Groups Comparisons: I believe the text call outs for Figure 4A and B are reversed. If so, please correct.
