# Supplementary material for: Hippocampal damage disrupts the latent decision-making processes underlying approach-avoidance conflict processing in humans
Source: PLoS Biol. 2025 Feb 11;23(2):e3003033. doi: 10.1371/journal.pbio.3003033 (PMC11849986; doi:10.1371/journal.pbio.3003033)
Supplement: Attachment — Submitted filename: Le Duc et al. 2024 PLoS Biol Response to Reviewers Final.docx [file pbio.3003033.s001.docx]

**Reviewer #1**

We were pleased to read that Reviewer 1 found merit in our work and that our reported findings are of interest.

1. “*Many statistical tests are reported but there appears to be no multiple comparison strategy - the p-values from these tests are therefore not interpretable in a strict sense. A statistically rigorous correction method should be implemented.*”

We thank Reviewer 1 for raising this important point and for giving us the opportunity to clarify our approach to multiple comparison correction.

We adopted conventional practice for studies involving multiple experiments (in our case, multiple tasks: AAC, Stroop, Go/No-Go). We did not correct for the number of tasks (i.e., n = 3) nor did we correct for the number of response measures within each task (e.g., Proportion of Approach and Response Time for AAC). Only a single LMM was fit for each response measure (although more than one test may have been used to assess model fit i.e., Chi-squared test, likelihood ratio test) and thus it was not necessary to correct for the number of main effects and interactions (as is the case, for instance, when more conventional omnibus ANOVAs are conducted). Importantly, however, all post-hoc pairwise comparisons to explore significant main effects and interactions were corrected for multiple comparisons using Tukey’s HSD procedure. This is noted in the statistical methods section and also made clear in the new statistical tables. Note that since the majority of our findings of interest were highly significant (p < 0.001), even if we were to deviate from conventional practice (i.e., correct for 3 experimental tasks) our conclusions would remain unchanged. Because of this, we have opted not to change our reporting of statistics in the current version of the manuscript. We can, however, provide both the original p-values, as well as p-values corrected for the number of experimental tasks, if the editor or reviewer feel that this is important to do.

Our reporting of hDDM findings also followed conventional practice. Only one single best fitting model was applied and this generated all the posterior distributions for the within- and between-group hypothesis tests. Discussion of similar issues regarding multiple comparisons corrections within hDDM (e.g., <https://groups.google.com/g/hddm-users/c/CI4ip3EnoE0/m/9LAUyln-BAAJ>) suggest that one “advantage” of modeling within a Bayesian framework is that the inclusion of more parameters tends to increase the model’s uncertainty about the value of any given parameter, resulting in a kind of natural “correction” for greater complexity. Thus, when highest-density intervals suggest a credible difference between parameters even under such circumstances, it suggests that such differences overwhelm the greater uncertainty that results from estimating a larger number of parameters (see reply by Michael Frank in the above-linked discussion, one of the creators of the hDDM package and a highly-regarded expert in computational modeling, for confirmation of this assertion).

2. “*The results are framed as relevant for mental health disorders in a wider sense (i.e. beyond the HC lesion group). The idea that approach-avoidance conflict tasks are relevant for anxiety disorders is occasionally made in original research papers, but despite 7 decades of research there is no evidence for this assumption beyond the fact that GABA agonists reduce behavioural cautiousness in these tasks and also reduce subjective anxiety in patients (see for reviews e.g. (Cryan and Sweeney, 2011; Ennaceur, 2014; Ennaceur and Chazot, 2016; Harro, 2018; Bach, 2022). Approach-avoidance conflict is surely an important economic setup (i.e. a mixed gamble) with relation to real-life scenarios, but the clinical claim is probably unwarranted.*”

We agree with Reviewer 1 on this point and have been more careful not to overstate our findings. We have now tempered our statement regarding the involvement of AAC in mental health disorders in the first paragraph of the Introduction and moreover, now state in the Discussion (page 37) that the relevance of our findings to mental health disorders, in particular anxiety, is unclear, citing a handful of the references highlighted by the reviewer.

3. “*It would be good if the authors could place their task within the wider space of human approach-avoidance conflict tests, which differ markedly.*”

In the same Discussion paragraph in which we address point 2, we now also briefly place our work within the wider context of human AAC.

4. “*Results are difficult to read. Perhaps some of the statistical minutiae could go into tables?*”

We thank the reviewer for this suggestion. All statistical details have now been moved into tables, which has also allowed us to address Reviewer 2’s request (Reviewer 2, point 4) for full details of individual statistical tests.

**Reviewer #2**

We were pleased to read that Reviewer 2 was of the opinion that we provide novel and convincing evidence that hippocampal damage impacts the latent decision-making processes during approach-avoidance conflict processing.

Major comments

1)	“ *p. 3, Introduction, 2nd para: Either here or in the Discussion (or both) it would be appropriate to cite work in nonhuman primates that suggests hippocampus damage produces a greater propensity to approach reward in the presence of threat (Y. Chudasama et al., 2008).*”

We thank the reviewer for recommending this citation. We have now cited Chudasama et al. (2008) both in the Introduction as well as the Discussion sections.

2)	“*Table 1. The value for Left HPC seems anomalous. How can this value be greater than the two values for aHPC and pHPC? Although the reader is directed to ref # 23 for details, it does not mention how HPC is calculated. Are aHPC and pHPC the same size as each other (so 'HPC' is simply the mean of aHPC and pHPC)? Please explain.*”

The HPC volume is the sum of the aHPC and pHPC volumes. In brief, the entire HPC was delineated using the guidelines in Pruessner et al. 2002, and then split into anterior and posterior portions using the uncal apex as the dividing landmark. Thus, depending on the participant, either the aHPC or pHPC may make up a greater portion of the entire HPC (or they could be similar in size), which is then reflected in the Z-score values as they are calculated with respect to the control group means. We now provide additional information in the legend for Table 2 regarding the delineation of the different regions.

3)	“*p. 17, Methods, end of 1st para: the authors describe the notation from their hierarchical Drift Diffusion Models (hDDMs) as though it can be read out as 'significant' or not. They are correct that capital P refers to the probability of overlap of the posterior distributions of two conditions, but I believe they are in error in assigning 'significance'. Interpreting the posterior probability (P) as a metric of statistical significance is antithetical to the Bayesian framework that underlies the hDDM. Moreover, this frequentist (mis)interpretation is particularly ill-advised because the hDDM does not always use the normal distribution (a key assumption in many Frequentist models) and instead utilizes non-normal distributions (e.g., Beta, Gamma) that are uncommon to most tests of statistical significance (see: Sequential Sampling Models — HDDM 0.9.8 documentation). It is more appropriate to interpret the P returned by the hDDM as the probability that the parameter estimate (e.g., Drift Rate) for one condition (e.g., Patients) is greater than that same parameter estimate from another condition. The authors should therefore remove mention of "significance" when discussing their hDDM results and instead interpret P values as the probability of superiority. Because the value of P will differ depending upon which posterior is used as the reference (see: Basic HDDM Tutorial — HDDM 0.9.8 documentation ), the authors should also edit their Results to explicitly mention which two posteriors are being compared. For example, p. 23 (1st sentence) could instead read as: "The control group drift rate was almost certainly lower/more negative relative to the patient group (P(Controls > Patients) = 0.0046) on [Conflict] trials...". Alternatively, if the authors prefer the Frequentist interpretation of statistical significance, they could remove all P values and instead use an estimation approach to hypothesis testing where the null hypothesis is rejected if the null value (e.g., 0) is not contained within the 95% highest density interval (HDI) of the posterior (see Kruschke, 2014).”*

We thank Reviewer 2 for raising this important point and concur that the use of frequentist language in the description of our hDDM findings is inappropriate given the Bayesian framework that underpins the hDDM. We had originally opted for the former approach because many psychologists and neuroscientists are more familiar with that framework and approach, though we recognize that doing so came at a cost of clarity and consistency with the Bayesian framework. As such, we have now replaced all frequentist terminology and have made clear, for each of our hypothesis tests, which two posterior distributions are being compared (see pages 26-27). To aid readers who are less familiar with the interpretation of hDDM output and Bayesian terminology, we have also provided additional detail in the Methods section (see page 17).

4) “*p. 19, para 2 under Decision phase: The authors report cohen's d for all three comparisons combined (e.g., "all d ≥ 1.64, d 90% CI all [1.57 3.70]"), but this is unhelpful. Instead, authors should report all three Cohen's d effect sizes (and all 3 corresponding t values). Similarly, authors should refrain from reporting minimum effect sizes (e.g., b values from linear models) when the effects are statistically significant.*”

In response to Reviewer 1 Point 3, we have now moved all statistical minutia to individual tables. As such, all individual effect sizes are now reported for all statistical tests.

5 & 6) “*p. 22, Results, under Within-Group Comparisons: The authors should avoid interpreting P values as p-values (see Comment #3, above). Instead, authors should report that P(No-Conflict > Conflict)Patients= 0.49. The report that, "all P ≤ 0.018" is also misleading because P actually refers to the probability (X > Y). Please revise this notation throughout.*”

“*p. 22, Results, under Between-Groups Comparisons: As an example of the misuse of notation described in #3 (above), the authors state "On both No-Conflict Positive and No-Conflict Negative trials, there was no indication that groups differed significantly in estimates for either non-decision time (both Ps => 0.26), drift rate (both Ps => 0.77), or threshold (both Ps => 0.72)." This is confusing because the authors do not clarify which group is higher/lower than the other. Here and elsewhere, please explicitly state which parameters are being compared and which group shows the greater relative probability of superiority.*”

As with Comment 3, all frequentist language has been replaced and we provide the directionality of all individual Within-Group and Between-Group comparisons.

7) ”*p. 29, first para: the idea regarding a role for the hippocampus in 'retrieval of evidence associated with undesirable outcomes' works for the present study and several others, but does not take into account the findings from studies in rodents and nonhuman primates that the conflict in approach-avoid conflict tasks can involve unlearned or innate threat (e.g., novel foods, snakes). For example, in rodent studies, ventral hippocampal damage disrupts innate avoidance of novel foods and entry onto open arms of a plus maze, which suggests a broader role for the hippocampus in resolving approach-avoid conflict. If so, this means the hippocampus is essential for not only conflict with recently learned items with conflicting valence, but also for some classes of innate ones. Indeed, it is worth discussing the point that the hippocampus plays an essential role not only in cases with learned 'cued' valences involving secondary reinforcers (points, as here) but also in cases in which approach-avoid conflicts appear to be based on innate (or genetically programmed) threats or rewards. These findings are more consistent with the authors' later statements (e.g., last sentence p. 29) that absence of a hippocampus may hamper the detection of motivational conflict.*”

We thank Reviewer 2 for this helpful comment. We have inserted additional text to address this point in the Discussion (see page 37).

Specific comments: minor

1) “*p. 12, Introduction, 2nd para: primate homolog of the *rodent* vHPC.*”

‘Primate’ has now been corrected to ‘rodent’.

2) “*p. 28, 2nd para; the authors state that the volume loss was 'predominantly within the anterior portion of the hippocampus'. From Table 2 it appears that the damage to aHPC was slightly greater than damage to the pHPC, but this does not strike me as 'predominantly' to aHPC. Please revise the statement or provide more information in support of the claim. Also, is it possible to run a correlation of the patient's approach scores against extent of aHPC damage? That might be more compelling.*”

We agree with Reviewer 2 here and have now revised our statement as suggested (page 34). We have refrained from running a correlation of the patients’ approach scores against extent of aHPC damage since our study is very much underpowered for such an analysis (8 patients) - a finding either way would be difficult to interpret with any confidence.

3) “*Figure 3: it is difficult to compare the amount of overlap in the Control and Patient plots in Fig 3 because the axes are different. Please make x-axis and y-axis the same for both plots within a panel. Also, the authors should consider separating the plots shown in Fig 3A-C (within-group comparisons) from those shown in D (between-group comparisons).*”

As suggested, the axis ranges are now identical across Control and Patient plots in Figure 3, and within-group and between-group comparison plots have been separated into two separate figures (new Figures 3 and 4).

4) “*p. 28, second para: it would be appropriate to cite the work in nonhuman primates here (see # 1 under Specific comments: major).*”

As noted above, the work in nonhuman primates has now also been cited here in the Discussion.

5) “*p. 28, second para, last sentence: the word 'human' is used twice unnecessarily. Please revise.*”

We have removed the extra use of the word ‘human’.

6) “*p. 30, second para: the authors discuss a possible role for the perirhinal cortex in approach-avoid conflict. First, the fMRI study in humans that implicates perirhinal cortex is correlative, so not a direct conflict. Although they view the one rodent study as conflicting with the current study, it seems that an alternative explanation for the rodent findings is that the study relied on two objects (combined) in the approach-avoid scenarios. Because the perirhinal cortex is implicated in learning about the conjunctions of objects (Saksida et al., 2007), it is possible that this aspect of the studies was responsible for the effects of the perirhinal cortex manipulations. It may also be worth noting (here or elsewhere) that the anterior hippocampus has different anatomical connections than the posterior hippocampus, including direct projections to the prefrontal cortex (Barbas and Blatt, 1995).”*

We thank Reviewer 2 for this input. Being mindful of the proposed role of the perirhinal cortex in learning object feature conjunctions, we actually used objects that possessed low feature overlap in our rodent perirhinal cortex approach-avoidance study (Dhawan et al., 2023). More specifically, we chose objects that the rodents were able to discriminate successfully in an oddity judgement paradigm (Dhawan et al., in preparation). As recommended, we now refer to the different connections of the v/aHPC and d/pHPC as well as their corresponding subfields, citing Barbas and Blatt (1995) and the more recent work of Dalton et al. (2019) in humans (bottom page 36).
