# Supplementary material for: Hippocampal damage disrupts the latent decision-making processes underlying approach-avoidance conflict processing in humans
Source: PLoS Biol. 2025 Feb 11;23(2):e3003033. doi: 10.1371/journal.pbio.3003033 (PMC11849986; doi:10.1371/journal.pbio.3003033)
Supplement: Attachment — Submitted filename: Le Duc et al. 2024 PLoS Biol Response to Reviewers 2.docx [file pbio.3003033.s003.docx]

**Reviewer #1**

We thank Reviewer 1 once again for their time and helpful thoughts. As suggested in their follow-up comment regarding correction for multiple comparisons, we have now added the following paragraph to the Methods section (manuscript bottom page 15):

“For all LMMs, we adopted a significance threshold of α = 0.05. In other words, we did not have an a priori strategy to account for multiple LMMs being conducted for multiple behavioral tasks and response measures (7 LMMs in total), thereby increasing the probability of Type I errors occurring. Adopting a stringent Bonferroni corrected α of 0.00714 (0.05/7) would result in 2 previously significant effects pertaining to the learning phase of the AAC task becoming trends (i.e., 0.007 < p < 0.0143; see Table 3) and 3 previously significant effects becoming non-significant (i.e., p > 0.0143). Two of these latter effects relate to interactions with RT during the decision phase of the AAC task (see Table 4) and the third relates to the effect of cue type on Go/No-Go task inhibition errors (see Table 6). None of these changes would impact the conclusions of this study. Critically, the effects that are central to the conclusions of this study survive Bonferroni correction. Specifically, participants demonstrated significant learning of stimulus valences across the learning phase (p < 0.001) (see Table 3) and at the decision phase, there was a significant group by condition interaction in the proportion of approach responses (p < 0.001), with a significant difference on Conflict trials (p < 0.001) but not No-Conflict Positive nor Negative trials (both p > 0.8) (see Table 4).”

Given the iterative nature of the Holm-Bonferroni approach and the overall structure of our analyses (multiple LMMs with different main and interaction effects tested), we felt that a Bonferroni correction was, in fact, the most appropriate approach here and hence, adjusted α by the number of LMMs (i.e., 7) that were run.

**Reviewer #2**

We thank Reviewer 2 for their positive comments and for picking up the error on the call-outs for Figures 4A and 4B – these have now been corrected (manuscript page 28).
