# Supplementary material for: Hippocampal damage disrupts the latent decision-making processes underlying approach-avoidance conflict processing in humans
Source: PLoS Biol. 2025 Feb 11;23(2):e3003033. doi: 10.1371/journal.pbio.3003033 (PMC11849986; doi:10.1371/journal.pbio.3003033)
Supplement: Attachment — Submitted filename: Le Duc et al. 2024 PLoS Biol Response to Reviewers 3.docx [file pbio.3003033.s004.docx]

All linear mixed model p-values described in the Results section have now been Bonferroni-corrected (i.e., p x 7). Similarly, Bonferroni corrected p-values have been added to all statistical tables. In order to maintain full transparency and to alleviate potential reader concerns with regards to Type II errors, we have kept the uncorrected p-values in the statistical tables. Notably, many of the uncorrected p-values for our key findings are so small that they remain at < 0.001 even after Bonferroni correction.

In light of these changes, we have also replaced the paragraph we had originally added to the Methods section in the last revision with:

“Lastly, since multiple LMMs were conducted across multiple behavioral tasks and response measures (seven LMMs in total), we accounted for the increased probability of Type I errors occurring by additionally adjusting all p-values using a Bonferroni correction (i.e., p x 7). Both uncorrected and corrected p-values are reported in the statistical tables (Tables 3 – 6).”
